# Artificial Intelligence in Microsurgical Planning: A Five-Year Leap in Clinical Translation

**DOI:** 10.3390/jcm14134574

**Published:** 2025-06-27

**Authors:** Omar Shadid, Ishith Seth, Roberto Cuomo, Warren M. Rozen, Gianluca Marcaccini

**Affiliations:** 1 Department of Plastic and Reconstructive Surgery, Peninsula Health, 2 Hastings Road, Melbourne, VIC 3199, Australia; omarshadid4@gmail.com (O.S.); ishithseth1@gmail.com (I.S.); warrenrozen@hotmail.com (W.M.R.); 2 Plastic and Reconstructive Surgery, Department of Medicine, Surgery and Neuroscience, University of Siena, 53100 Siena, Italy; robertocuomo@outlook.com

**Keywords:** microsurgery, artificial intelligence, machine learning, augmented reality, surgical planning, preoperative, intraoperative, postoperative

## Abstract

**Background:** Microsurgery is a highly complex and technically demanding field within reconstructive surgery, with outcomes heavily dependent on meticulous planning, precision, and postoperative monitoring. Over the last five years, artificial intelligence (AI) has emerged as a transformative tool across all phases of microsurgical care, offering new capabilities in imaging analysis, intraoperative decision support, and outcome prediction. **Methods:** A comprehensive narrative review was conducted to evaluate the peer-reviewed literature published between 2020 and May 2025. Multiple databases, including PubMed, Embase, Cochrane, Scopus, and Web of Science, were searched using combinations of controlled vocabulary and free-text terms relating to AI and microsurgery. Studies were included if they described AI applications during the preoperative, intraoperative, or postoperative phases of microsurgical care in human subjects. **Discussion:** Using predictive models, AI demonstrated significant utility in preoperative planning through automated perforator mapping, flap design, and individualised risk stratification. AI-enhanced augmented reality and perfusion analysis tools improved precision intraoperatively, while innovative robotic platforms and intraoperative advisors showed early promise. Postoperatively, mobile-based deep learning applications enabled continuous flap monitoring with sensitivities exceeding 90%, and AI models accurately predicted surgical site infections, transfusion needs, and long-term outcomes. Despite these advances, most studies relied on retrospective single-centre data, and large-scale, prospective validation remains limited. **Conclusions:** AI is poised to enhance microsurgical precision, safety, and efficiency. However, its integration is challenged by data heterogeneity, generalisability concerns, and the need for human oversight in nuanced clinical scenarios. Standardised data collection and multicentre collaboration are vital for robust, equitable AI deployment. With careful validation and implementation, AI holds the potential to redefine microsurgical workflows and improve patient outcomes across diverse clinical settings.

## 1. Introduction

Microsurgery is a technically demanding field within the domain of plastic and reconstructive surgery, encompassing free-flap transfers, peripheral nerve repairs, limb replantations, and composite tissue allotransplantations [1,2,3]. The success of surgical procedures is contingent on meticulous preoperative planning, dexterous intraoperative techniques performed under magnification, and diligent postoperative monitoring [4,5]. In recent years, artificial intelligence (AI) has garnered significant attention to enhance each stage [6,7]. AI methodologies, including machine learning (ML), deep learning, computer vision, and augmented reality, present opportunities to analyse complex medical data, assist in decision-making processes, and provide direct aid in surgical tasks [8]. Notably, initial reviews within plastic surgery have identified an expanding role for AI in diagnostics, surgical planning, intraoperative guidance, and outcome prediction [9,10]. Since 2020, there has been a notable increase in studies that apply AI specifically to microsurgery, indicating the field’s receptiveness to innovation.

The evolution of AI coincides with a broader shift towards digital health integration, precision medicine, and data-driven surgical decision-making [6]. Due to their complexity and variability, microsurgical procedures offer an ideal platform for testing and adopting AI-driven technologies [7]. Surgeons are increasingly required to interpret large volumes of imaging data, make time-sensitive intraoperative decisions, and ensure early detection of complications. AI tools can support these functions through real-time analysis, pattern recognition, and predictive modelling [8]. Integrating AI into microsurgical workflows may offer a transformative paradigm shift as healthcare systems strive for improved outcomes, reduced complications, and cost efficiency. Furthermore, the rising availability of high-quality surgical data and improved algorithm training has made it feasible to develop clinically useful, task-specific AI systems tailored to microsurgical needs.

This narrative review examines the applications of AI in microsurgery from 2020 to 2025. We synthesise the findings from various subspecialties, including breast reconstruction, head and neck reconstruction, and limb replantation, to provide a comprehensive overview of the influence of AI on microsurgical practice. The review is organised according to the operative timeline, encompassing preoperative, intraoperative, and postoperative phases, while also addressing the integration of clinical workflows, current challenges, and future prospects. Our objective is to critically assess the existing scientific knowledge and offer insights into how emerging AI tools can enhance microsurgical precision, improve patient outcomes, and mitigate longstanding challenges within the field.

## 2. Materials and Methods

A comprehensive literature search was conducted to capture AI’s evolution and clinical translation in microsurgical practice from 2000 to 1 May 2025. We queried PubMed/MEDLINE, Embase, the Cochrane Central Register of Controlled Trials (CENTRAL), Scopus, and Web of Science utilising controlled vocabulary (MeSH or EMTREE) and free-text terms. The primary search terms included “artificial intelligence,” “machine learning,” “deep learning,” “computer vision,” and “augmented reality;” these were combined using Boolean operators to include “microsurgery,” “free flap,” “perforator flap,” “nerve repair,” “replantation,”, “perforator mapping,” “intraoperative guidance,” “flap monitoring,” “postoperative surveillance,” and “outcome prediction.” Additionally, we performed hand searches of the reference lists from significant reviews and clinical guidelines.

The inclusion criteria were peer-reviewed original research articles, systematic reviews, meta-analyses, and clinical trials published in English that described AI algorithms or AI-enabled technologies applied to any phase of microsurgical care (preoperative planning, intraoperative assistance, or postoperative monitoring) in human subjects. The exclusion criteria included non-English publications, abstracts, or conference posters without full text; animal or bench-top studies without clinical correlation; and articles focused exclusively on general surgical AI applications outside the microsurgical domain.

## 3. Discussion

### 3.1. Preoperative Applications

#### 3.1.1. AI for Perforator Mapping and Surgical Planning

Preoperative planning in microsurgery often requires identifying suitable blood vessels (perforators) and evaluating patient anatomy using imaging techniques. AI-driven image analysis has demonstrated considerable potential in automating and enhancing these processes. A summary of the key studies supporting these applications is presented in Table 1. For example, Mavioso et al. (2020) introduced a computer vision algorithm aimed at detecting perforator vessels in CT angiography (CTA) specifically for DIEP flap breast reconstruction [11]. In a pilot study involving 40 patients, their software consistently identified perforators and estimated vessel calibre, reducing the time required for radiologists to delineate perforators by approximately two hours per case. Similarly, Shen et al. (2022) integrated U-net deep learning with CTA and ultrasonography to accurately localise perforators for anterolateral thigh flaps, accurately identifying target vessels for flap harvest [12]. This was further corroborated by De La Hoz et al. (2023), who achieved over 92% accuracy in automatically identifying perforator “hot spots,” matching Doppler-confirmed locations [13]. Their approach provided a radiation- and contrast-free alternative for perforator mapping, thereby reducing reliance on CTA and potentially streamlining preoperative flap planning.

Furthermore, Saxena et al. (2022) demonstrated that AI can accurately segment vascular trees on synthetic CTA-like images, achieving a sensitivity and specificity of over 93% [22]. Their models processed each frame in under 0.2 s, suggesting the feasibility of near real-time perforator mapping in clinical practice. Although trained on synthetic datasets, these results provide a robust proof of concept for AI-driven vessel segmentation. These advancements indicate that AI can augment preoperative imaging interpretation, thereby assisting surgeons in efficiently locating optimal donor vessels and planning flap design.

In addition to vessel mapping, AI has been employed to interpret intricate imaging data for microsurgical planning. Lim et al. (2024) evaluated large-language model AI tools for analysing CTA scans in the context of DIEP flap planning [19]. In their investigation, an AI-generated summary of CTA findings was juxtaposed with reports from expert radiologists. AI demonstrated proficiency in accurately summarising significant anatomical details but failed to capture nuanced information that seasoned surgeons considered critical. This highlights both the potential and existing limitations of AI “virtual assistants” in preoperative evaluation. At the same time, they may swiftly synthesise routine imaging features. Still, the necessity of human oversight persists to identify subtle yet essential details (e.g., vessel quality and anatomical variants) crucial for surgical planning.

Beyond algorithmic performance, the clinical value of AI-driven perforator mapping depends on its integration into existing imaging workflows. Standalone solutions necessitate export and manual upload to proprietary AI software, followed by the reimport of annotated datasets back into the reporting suite [23]. This method may introduce workflow latency and place a burden on users. In contrast, vendor-agnostic plugins or native modules within platforms provide vessel-detection overlays directly in the CTA review window, eliminating the need for data transfer and minimising disruptions to radiology and surgical planning processes [24]. Cloud-based services additionally enable near real-time analysis without requiring local hardware investment, albeit with increased demands for secure data transmission and compliance with institutional and regulatory safeguards [25]. Each deployment model comes with trade-offs: embedded solutions reduce training requirements and IT overhead, whereas standalone or cloud tools enable iterative feature enhancements and advanced analytics at the cost of additional integration steps [26]. To realise the full potential of AI in microsurgical planning, collaboration among surgical teams, radiologists, and information technology specialists is vital to establish robust data flow protocols, enforce image security measures, and codify standard operating procedures that align AI outputs with multidisciplinary planning conferences.

#### 3.1.2. Patient Selection and Outcome Prediction

Preoperative risk stratification represents an area where AI has made significant contributions. Machine learning models have been developed using extensive clinical datasets to predict the likelihood of microsurgical complications in patients, thereby facilitating surgical decision-making and informed consent discussions. In the context of head and neck reconstruction, Asaad et al. (2023) implemented machine learning algorithms based on patient factors to forecast the failure of free flaps and associated complications [14]. Their models demonstrated moderate accuracy, with reported performance ranging from 63% to 98% in predicting flap loss, depending on the specific algorithm and outcome endpoint used. Furthermore, they identified key risk predictors, including patient gender, smoking status, utilisation of vein grafts, and comorbidities such as hypertension. These predictors, many of which corroborate established clinical risk factors, are amenable to automatic weighting by AI, enabling the generation of individualised risk profiles. Formeister et al. (2020) utilised machine learning to predict complications in head and neck free tissue transfers, finding that data-driven decision trees could effectively stratify patients based on their likelihood of experiencing significant postoperative events [17]. In microsurgical breast reconstruction, extensive retrospective analyses have yielded machine learning models capable of identifying patients at risk for flap failure. O’Neill et al. (2020) evaluated over 1000 free-flap breast cases, of which 12 (approximately ≈ 1%) resulted in total flap loss [21]. They identified that an AI model could highlight obesity and active smoking as prominent predictors of failure factors already recognised by surgeons but delineated and weighted through an unbiased algorithmic methodology here. An additional study by Myung et al. (2021) focused on donor-site morbidity in abdominal flap breast reconstruction [20]. Utilising a cohort comprising 568 patients, they validated that machine learning classifiers could predict donor-site complications, such as abdominal wound issues, with approximately 81% accuracy.

While these prognostic models have yet to become standard practice, they illustrate the potential for AI to enhance preoperative patient selection. For instance, they may assist surgeons in optimising patients’ risk factors before surgery or consider alternative reconstructive strategies for individuals classified as high-risk. Notably, these models continually improve as they undergo training on larger datasets and can be updated as new outcomes are observed, thereby increasing their accuracy over time.

Virtual Surgical Planning and Patient Education: AI is explored in virtual surgical planning simulations and patient counselling before microsurgery. Neural networks have been utilised to create three-dimensional predictions of postoperative appearances [16]. For example, Chartier et al. (2022) developed BreastGAN, a generative adversarial network trained to produce patient-specific visualisations of expected reconstruction outcomes [16]. By quantitatively comparing simulated and actual postoperative images using structural similarity index (SSIM) and peak signal-to-noise ratio (PSNR), they reported high fidelity between predictions and reality. These simulations improved patient understanding of likely aesthetic results and reduced decisional conflict scores by 25% in a randomised vignette study.

Another study by Jeong et al. (2023) evaluated ChatGPT-3.5 against Google Search for answering 50 common patient questions about free-flap surgery [18]. ChatGPT’s answers significantly outperformed Google’s responses in accuracy, completeness, and readability. This demonstrates AI’s ability to deliver patient-centred education. Similarly, Berry et al. (2024) surveyed 120 plastic surgeons and 150 patients to compare AI-generated microsurgical educational materials (via GPT-4) against traditional brochures and web resources [15]. Notably, 88% of surgeons and 75% of patients indicated they would preferentially use AI-generated materials for preoperative counselling, further underscoring the role of AI’s potential for patient education in microsurgery.

### 3.2. Intraoperative Applications

#### 3.2.1. AR Navigation

Intraoperative AR technology has significantly advanced in microsurgery, providing surgeons with enhanced visualisation capabilities by superimposing digital information onto the operative field. A notable application of this technology is the AR-assisted harvesting and insertion of osseous flaps. Pietruski et al. (2020) conducted a proof-of-concept study utilising AR to guide fibula free-flap harvest in mandibular reconstruction [27]. In this study, surgeons utilised a heads-up display that projected a virtual cutting guide alongside the planned bone segments onto the patient’s fibula. Remarkably, the AR system facilitated osteotomies with an accuracy comparable to that of traditional 3D-printed cutting guides; angular and distance deviations remained within a few degrees and millimetres of the virtual plan, with no clinically significant discrepancies in precision. This suggests that AR could potentially supplant expensive patient-specific guides in the future, thereby enhancing flexibility (as the surgical plan can be adjusted in real time) and reducing preparation time.

Another study revealed that the use of AR smart glasses during microsurgery improved the ergonomics and visualisation of the operative field for the operators [28]. Falola et al. (2024) described an AR microscope setup that projects digital microscope images onto a visor, enabling surgeons to operate with head-up visualisation [28]. Trainees utilising this system reported enhanced depth perception and reduced fatigue, suggesting the potential value of AR in microsurgical education. While the application of AR technology in microsurgery is still in its nascent stages, these instances underscore its potential to provide real-time navigational assistance—whether for bone alignment, vessel identification, or simply minimising the surgeon’s need to divert attention from the operative field—ultimately striving to enhance intraoperative efficiency and accuracy.

#### 3.2.2. Intraoperative Perfusion Assessment and Assistance

AI has also been utilised intraoperatively to evaluate tissue perfusion and inform surgical decision-making. A summary of the key studies supporting these applications is presented in Table 2. A notable application involves the employment of AI to interpret indocyanine green (ICG) angiography videos [29]. Singaravelu et al. (2024) developed a machine learning model to analyse intraoperative ICG video sequences and objectively recommend which flap portions demonstrate compromised perfusion. They should therefore be excised [29]. Trained on the judgments of expert surgeons, the AI model achieved an accuracy exceeding 99% in test videos when classifying flap areas as adequately perfused (“keep”) versus poorly perfused (“excise”). Notably, the algorithm established a threshold for fluorescence intensity (approximately 22 grayscale units), below which tissue is likely to be ischemic and needs debridement. This proof-of-concept demonstrates that AI can replicate the intraoperative decision-making abilities of an experienced microsurgeon in assessing perfusion. With further validation, such tools can enhance surgeons’ judgment, particularly in borderline cases, thereby potentially preventing insufficient and excessive flap tissue debridement.

Intraoperative AI is not restricted solely to perfusion analysis. Other research teams have investigated the potential of AI to offer real-time surgical guidance and automation [30]. For instance, a study by Atkinson et al. (2024) involved experimenting with applying large language models as on-demand advisors in the operating room [30]. The study presented various intraoperative crisis scenarios, such as a sudden arterial thrombosis in the flap, to evaluate its capacity and suggest appropriate management steps. The responses provided by the AI were generally factually accurate, similar to the reactions one might expect from a senior resident, yet they lacked case-specific nuance. This observation suggests that while AI can reinforce standard principles, it cannot replace the contextual judgment of a skilled microsurgeon. Furthermore, computer vision algorithms have been effectively utilised to recognise surgical instruments and structures within the operative field. Nakazawa et al. (2020) developed a real-time video analysis system employing a convolutional neural network to detect the microsurgical needle tip during suturing [32]. This capability lays the groundwork for AI-augmented surgical microscopes that could overlay needle trajectory guidance, quantify tremor, and issue safety alerts when the needle approaches critical structures, ultimately enhancing microsurgical precision and ergonomics.

Similarly, Koskinen et al. (2022) established a deep learning system that automatically tracks instrument motion and the surgeon’s hand movements, thereby enabling a quantitative assessment of microsurgical technique and coordination [31]. These technological advancements lay the foundation for future automation or AI-enhanced control of surgical instruments; for example, a robotic system capable of intelligently stabilising a blood vessel or a microscope that automatically focuses and centres based on instrument detection.

Despite promising accuracy in pilot studies, AI-based perfusion assessment tools face significant validation challenges before they can be routinely adopted in clinical practice. Most models are trained on narrow datasets—often a single flap type, such as DIEP or ALT—using standardised ICG dose, imaging hardware, and acquisition angles [29]. When applied to other flap varieties (e.g., muscle, fasciocutaneous, and composite), differences in tissue thickness, intrinsic fluorescence uptake, and vascular architecture can shift the fluorescence signal and undermine threshold-based algorithms [33]. Intraoperative variables, including ambient lighting, camera positioning, and surgeon manipulation, introduce additional noise not represented during model training [33].

Therefore, to demonstrate true generalisability, these systems require multicentre, prospective validation across a spectrum of flap types, ICG protocols, and imaging platforms. Fluorescence thresholds should be recalibrated for each flap’s unique perfusion kinetics and benchmarked against gold-standard measures (e.g., laser Doppler flowmetry or direct microvascular probes). Clear reporting of sensitivity, specificity, and the rationale for chosen thresholds will be essential. Only through systematic, cross-institutional trials can AI perfusion tools earn the broad trust necessary for critical, real-time intraoperative decision-making.

In conclusion, intraoperative AI applications have improved visualisation, precision, and decision support. Augmented reality and robotics directly assist the surgeon’s hands, while AI algorithms analyse visual data and propose actions. Collectively, these tools signify a revolution in the microsurgeon’s intraoperative capabilities that extend beyond traditional loupes and intuition, by integrating digital intelligence to facilitate safer and more efficient surgical procedures.

### 3.3. Postoperative Applications

#### 3.3.1. Flap Monitoring with AI

The postoperative monitoring of free flaps is crucial in the context of microsurgery, as the early detection of vascular compromise can prevent flap failure. Traditionally, flap monitoring has depended on clinical examinations, which involve the assessment of skin colour, temperature, and capillary refill, or on basic technologies such as handheld Doppler probes. In recent years, AI-driven approaches have emerged, facilitating continuous; objective; and, in certain instances, remote flap monitoring. A notable innovation is the introduction of smartphone-based flap monitoring systems. Kim et al. (2024) reported on an AI-powered mobile application that evaluates photographs of flaps to identify signs of arterial or venous insufficiency [34]. Their deep learning models achieved a sensitivity of 97.5% for identifying arterial occlusion and a sensitivity of 92.8% for venous occlusion. Such a high level of sensitivity achieved from simple photographs is remarkable, as it approaches the accuracy of experienced nursing staff conducting serial examinations, all while being continuous and objective. The implications of this advancement suggest that an automated application could alert clinicians to potential issues even before they are visible to the human eye or during periods when staff are not physically present. In practical terms, this could mean that a flap exhibiting signs of developing venous congestion at 3 AM could trigger a smartphone alert, prompting immediate evaluation.

Similarly, Hsu et al. (2023) developed a deep learning integrated iOS application for free flaps that quantifies subtle colour changes associated with venous congestion [35]. Their diagnostic study at Chang Gung Memorial Hospital demonstrated that the application’s algorithm could accurately differentiate between normal flaps and those that are congested, providing quantified perfusion indices that correlate with clinical judgement. These tools not only mitigate the burden of continuous monitoring by staff but also standardise what was previously a subjective assessment.

In addition to visual monitoring, AI has been applied to other monitoring modalities. Research is ongoing to continuously explore the use of wearable sensors and machine learning in tracking parameters such as temperature, tissue oxygenation, and even biochemical markers in flaps. For example, investigators have explored the use of implantable Doppler signals, interpreted by machine learning (ML) algorithms, to reduce false alarms in perfusion monitoring [36]. Maktabi et al. (2025) integrated hyperspectral imaging (HSI) with convolutional neural networks to identify postoperative free-flap malperfusion within 72 h of surgery [36]. The study found a sensitivity of 70% and a specificity of 76%, demonstrating that HSI combined with AI could significantly enhance early detection of compromised flaps and potentially improve salvage rates [36].

Despite high sensitivity in pilot studies, smartphone-based flap monitoring faces several real-world hurdles. First, the upfront costs of developing, validating, and maintaining a medical-grade mobile app, along with ongoing software updates and user support, can be substantial, particularly for smaller centres [37]. Second, device variability (including different smartphone models, camera resolutions, and lighting conditions) may affect image quality and algorithm performance, necessitating rigorous, site-specific calibration [38]. Third, these applications require formal regulatory clearance (e.g., FDA and CE marking) and alignment with data-protection regulations, often involving lengthy legal and cybersecurity reviews [39]. Finally, integration with hospital IT and electronic medical record systems is rarely seamless, and there is currently no established reimbursement pathway for AI-assisted monitoring, which can slow adoption despite its clear clinical promise.

While not yet widespread, these studies foreshadow a near future in which flap monitoring is augmented by “smart” systems that continuously learn a patient’s baseline and flag anomalies instantaneously, potentially allowing salvage interventions to be initiated even earlier than human observation alone.

#### 3.3.2. Outcome Prediction and Quality Assessment

The postoperative phase encompasses immediate outcomes, longer-term results, and potential complications. AI methodologies have been utilised to forecast and elucidate these outcomes, often intersecting with the preoperative risk models previously discussed. A summary of the key studies supporting these applications is presented in Table 3. For example, ML algorithms have been trained on surgical databases for postoperative analysis to identify risk factors for complications such as wound infection, fat necrosis, or hospital readmission following microsurgery. A previous study pioneered an artificial neural network (ANN) model to predict surgical site infections after head and neck free-flap surgery, demonstrating that the AI outperformed traditional logistic regression in identifying high-risk patients [40]. Furthermore, Tighe et al. (2022) applied ML to identify risk factors for head and neck free-flap success, thereby providing a more sophisticated methodology for surgeons to benchmark their performance and detect deviations in outcomes at an early stage [41].

Moreover, a study conducted by Puladi et al. (2023) employed an ML model to predict which patients undergoing head and neck free-flap procedures would necessitate blood transfusions [42]. Although this outcome may seem peripheral, it is crucial for resource allocation and patient blood management. By incorporating patient and operative variables into their model, they could stratify patients based on transfusion risk, potentially guiding preoperative blood product preparation and intraoperative management.

These predictive models enhance postoperative care by guiding the intensity of surveillance and personalised follow-up. A patient flagged by an AI model as being at high risk for flap failure or major complications may be monitored in the ICU for a longer period or seen in the clinic more frequently. In contrast, a low-risk patient might safely undergo streamlined recovery protocols. In rehabilitation contexts, AI is also beginning to play a role.

In summary, the postoperative landscape of microsurgery is being reshaped by AI through enhanced monitoring and predictive analytics. From the critical first 48 h of flap monitoring to long-term functional recovery and transplant surveillance, AI tools augment clinicians’ ability to detect issues early and tailor follow-up to individual patient risks. While limited in prospective trials, the evidence to date consistently demonstrates AI’s high sensitivity in monitoring tasks and its capability in analysing big data for outcome trends. This is promising for improving microsurgical success rates, provided these tools are integrated thoughtfully into care pathways.

### 3.4. Challenges and Future Directions

Despite its potential, integrating AI into microsurgery presents significant challenges and limitations that warrant acknowledgment. Foremost among these are issues related to data quality, quantity, and representativeness. Machine learning algorithms, which require intensive training, necessitate substantial datasets comprising images or cases to achieve a high level of accuracy. In the context of microsurgery, compiling such datasets proves to be a challenging task. Procedures such as free flaps or replantations are complex and less prevalent than general surgical procedures, which restricts the data available from any single centre. Numerous AI models reported (e.g., for flap monitoring or outcome prediction) have been trained on retrospective, single-institution datasets that may lack generalisability. There is a risk that an AI tool may perform adequately within the environment where it was developed (e.g., an application trained predominantly on images of flap procedures from primarily light-skinned patients may exhibit reduced accuracy on darker skin due to differing colourimetric properties). Therefore, ensuring diverse and extensive training data, potentially through multicentre collaborations, is crucial for developing robust AI systems in microsurgery.

Additionally, issues concerning data quality, such as inconsistent documentation, missing data, or subjectivity in outcome definitions, may lead to a phenomenon known as “garbage in, garbage out”: the AI may learn erroneous or biased correlations. For instance, if one surgical team documents “partial flap failure” differently than another, an algorithm may misconstrue the outcomes. Standardising data collection in microsurgery (potentially through specialty society registries that contribute to AI research) will assist in mitigating this issue.

#### 3.4.1. Future Directions

**Figure 1:**
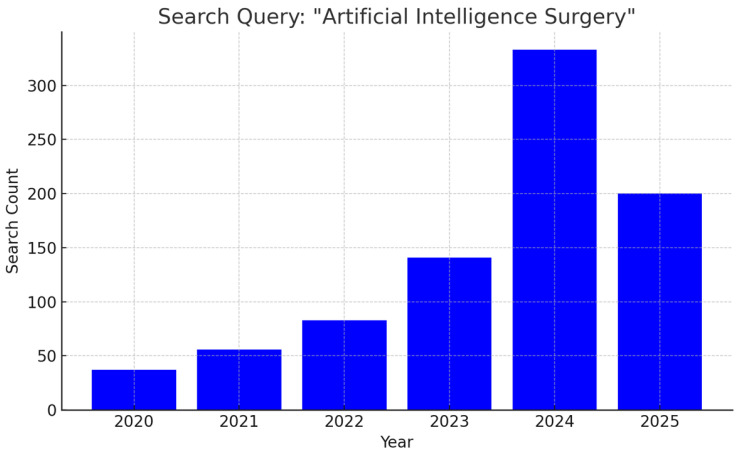
Bar graph illustrating the year-by-year growth in peer-reviewed articles addressing applications of artificial intelligence in surgical practice.

The past five years have witnessed an exponential increase in scholarly output examining AI applications in surgery, reflecting both technological advancements and growing clinical enthusiasm. From just 37 publications in 2020, the annual count surged to 141 in 2023 and soared above 300 in 2024, with 200 papers already in 2025 (Figure 1). As research transitions from proof-of-concept studies to early clinical trials, multidisciplinary collaborations between surgeons, data scientists, and device manufacturers are laying the groundwork for next-generation operating rooms where AI augments every step of the surgical journey.

Looking ahead, the convergence of AI and robotic platforms promises to transform microsurgical practice. Autonomous suturing systems, such as the Smart Tissue Autonomous Robot (STAR), have demonstrated precise suture placement in soft-tissue models, suggesting a path toward AI-driven microvascular anastomosis under surgeon supervision [43]. Reinforcement-learning algorithms may further enable real-time adjustment of instrument forces and trajectories, thereby reducing tremor and fatigue during lengthy procedures [44]. Beyond robotics, digital-twin models of patient anatomy—integrating preoperative imaging, biomechanical simulations, and intraoperative data streams—could allow for the virtual rehearsal of complex reconstructions, optimising flap design and pedicle orientation before the first incision [45]. Finally, AI-powered tele-mentoring systems may extend specialist expertise to remote or resource-limited settings, with real-time guidance and automated feedback on procedural steps [46].

In summary, the landscape of AI applications in microsurgery is rapidly expanding. CNNs trained on CTA and hyperspectral images have demonstrated high (>90%) concordance with Doppler gold standards for perforator mapping [11,13] (Table 4). Deep-learning methods, such as attention-UNet, further refine microvascular landmark detection [12], while computer vision models reliably segment complex vascular trees on CTA [22]. In parallel, ML and ANN models using clinical and demographic inputs achieve up to 95% accuracy in predicting free-flap complications and failures [14,17], and CUSUM-augmented ML has optimised real-time performance benchmarking [41] (Table 4). Generative AI frameworks like BreastGAN offer simulated aesthetic outcomes to enhance patient counselling [16], and LLMs such as ChatGPT deliver high-fidelity answers to patient queries about flap surgery [18]. Emerging modalities—including hyperspectral imaging coupled with CNNs show promise for early malperfusion detection [36], though all tools remain at the single-centre or pilot stage (Table 4). Together, these studies lay the groundwork for robust, multicentre validation of AI-augmented microsurgical planning and monitoring.

#### 3.4.2. Regulatory and Ethical Considerations

Despite these innovations, substantial regulatory and ethical barriers remain. In the United States, AI software classified as a medical device must navigate the FDA’s AI/ML SaMD Action Plan, including rigorous validation of continual learning algorithms and post-market performance monitoring [47]. Ethically, ensuring informed patient consent for AI-assisted interventions requires clear communication of algorithmic roles and limitations. Algorithmic bias, stemming from unrepresentative training datasets, presents risks of unequal outcomes across skin tones, ages, or comorbidity profiles, necessitating ongoing fairness audits and clear reporting of model performance broken down by key demographic variables [48]. Governance frameworks, such as the WHO’s Ethics and Governance of AI for Health guidelines, recommend multidisciplinary oversight committees and robust data-governance policies to safeguard patient rights and public trust [49].

Our review was restricted to English-language publications and omitted preclinical investigations, which may introduce selection bias and limit the comprehensiveness of our findings. Language restrictions can skew results by overrepresenting studies from Anglophone regions, potentially overlooking relevant data published in other languages. Similarly, excluding preclinical and animal model research may omit valuable mechanistic insights and early-stage validation of AI algorithms, thereby delaying the recognition of emerging technologies that have yet to reach clinical trials. Future work should consider multilingual searches and the incorporation of translational studies to provide a more holistic appraisal of AI applications in microsurgery.

## 4. Conclusions

AI has rapidly transitioned from a theoretical concept to a tangible contributor in microsurgery over the past few years. This narrative review has highlighted that AI applications now permeate all phases of microsurgical care—from preoperative imaging and risk assessment to intraoperative guidance with AR/robotics and to postoperative monitoring and outcome prediction. The collective evidence suggests that when appropriately applied, AI can enhance surgical precision, provide decision support, and potentially lead to better patient outcomes.

## Figures and Tables

**Table 1:** Summary of key artificial intelligence applications in preoperative microsurgery.

Study (Year)	Study Site	Application	Performance
Asaad (2023) [14]	Single Centre	Free-flap failure risk prediction	Machine learning models have identified smoking habits, the type of flap used, and the presence of vein grafts as the primary predictors of complications associated with flaps.
Berry (2024) [15]	Single Centre	Survey on AI-generated microsurgical education materials	Surgeons (88%) and patients (75%) preferred AI content for clarity and comprehensiveness.
Chartier (2022) [16]	Single Centre	Generative AI (BreastGAN) to simulate DIEP flap outcomes	Improved patient understanding of likely aesthetic results.
De La Hoz (2023) [13]	Single Centre	Convolutional neural network to assist with the mapping of abdominal perforators	High confidence localisation; pilot dataset showed >90% concordance with Doppler, suggesting the potential for a radiation-free, contrast-free mapping for flap planning.
Formeister (2020) [17]	Single Centre	ML to predict complications in H&N free flaps	Identified predictors, including flap type, patient comorbidities, and operative time; support targeted monitoring; and informed consent.
Jeong (2023) [18]	Single Centre	ChatGPT vs. Google for patient FAQs about free-flap surgery	ChatGPT achieved 92% accuracy/89% completeness vs. Google’s 78%/70%, at lower reading levels; patients rated ChatGPT’s clarity 85% vs. 55%.
Lim (2023) [19]	Single Centre	LLM to generate automated image–report summarisations of CTAs	LLMs correctly listed major vessels but missed nuanced variants ~30% of the time—valid for rapid summaries, not to replace experts.
Mavioso (2020) [11]	Single Centre	Computer vision algorithm for DIEP flap perforator detection	Reduced mapping time from ~2–3 h to 30 min per patient with millimetric accuracy—promises standardised planning.
Myung (2021) [20]	Single Centre	ML for donor-site complication prediction	Neural network achieved 81% accuracy in complication prediction—enabling targeted preoperative counselling and risk mitigation.
O’neill (2020) [21]	Single Centre	ML for DIEP flap failure risk prediction	Highlighted obesity and comorbidities as key risk factors, informing preoperative counselling
Saxena (2022) [22]	Single Centre	Convolutional neural network to assist with CTA vascular tree segmentation	Demonstrated that CNNs can robustly identify complex vessel patterns in CTA-like images, validating the feasibility of AI-based perforator mapping.
Shen (2022) [12]	Single Centre	Implement deeply supervised attention UNet for perforator localisation in ALT flap planning	Outperformed manual methods in accuracy and consistency, reducing subjectivity in perforator selection

H&N: Head and Neck; CTA: CT Angiography; DIEP: Deep Inferior Epigastric Perforator; LLM: Large Language Models; ML: Machine Learning; CNN: Convoluted Neural Network.

**Table 2:** Summary of key artificial intelligence applications in intraoperative microsurgery.

Study (Year)	Study Site	Application	Performance
Atkinson (2024) [30]	Single Centre	Assess LLM performance for intraoperative DIEP flap queries	Generated correct standard algorithms but lacked patient-specific nuance, paralleling resident knowledge, best used as a structured checklist.
Falola (2024) [28]	Single Centre	Microsurgical field visualisation in live procedures	Demonstrated feasibility of overlaying critical anatomical landmarks onto the operative view, improving spatial awareness and potentially reducing dissection time and errors.
Koskinen (2022) [31]	Single Centre	Develop a deep-learning system for automatic microsurgical tool detection and eye–hand coordination monitoring	Achieved >95% precision in classifying 6 instrument types and quantified gaze–hand alignment metrics, enabling objective intraoperative skill assessment and potential real-time guidance.
Nakazawa (2020) [32]	Single Centre	Microsurgical suturing	Real-time needle-tip tracking with 95% accuracy. Potential to issue safety alerts when the needle approaches critical structures.
Pietruski (2020) [27]	Single Centre	Fibula free-flap osteotomies	Achieved ≤3° angular error and ≤2 mm positional error vs. 3D guides; improved osteotomy precision and vessel safety.
Singaravelu (2024) [29]	Single Centre	Intraoperative perfusion assessment	Achieved >99% accuracy in classifying “keep vs. excise” regions; defined objective fluorescence thresholds for flap trimming

DIEP: Deep Inferior Epigastric Perforator; LLM: Large Language Models.

**Table 3:** Summary of key artificial intelligence applications in postoperative microsurgery.

Study (Year)	Study Site	Application	Performance
Hsu (2023) [35]	Single Centre	Early venous congestion detection	Demonstrated ~95% accuracy distinguishing normal from congested flaps, offering a low-cost monitoring modality
Kim (2024) [34]	Single Centre	Free-flap perfusion monitoring	Achieved 97.5% sensitivity for venous and 92.8% for arterial compromise; zero missed events—may drastically reduce nursing burden
Maktabi (2025) [36]	Single Centre	Combine HSI with CNN classifiers to detect postoperative free-flap malperfusion	Suggests HIS + AI could enhance early malperfusion detection and salvage.
Puladi (2023) [42]	Single Centre	Blood transfusion requirement	Achieved 78% stratification accuracy, enabling risk-adjusted patient blood management
Tighe (2022) [41]	Single Centre	Free-flap success benchmarking	Improved sensitivity and specificity of CUSUM charts in detecting performance deviations, enabling earlier identification of issues and targeted quality improvement

CUSUM: Cumulative Sum (Control Chart); ANN: Artificial Neural Network; HSI: Hyperspectral Imaging; CNN: Convoluted Neural Network.

**Table 4:** This table summarises key AI-driven tools applied to microsurgical flap planning and monitoring.

Tool/Model	Input Data	Clinical End Point	Key Studies	Validation Status
CNN for perforator mapping	CTA, HSI	Perforator localisation	De La Hoz et al. (2023) [13]; Mavioso et al. (2020) [11]	Pilot studies (>90% concordance vs. Doppler)
Deep vision/CV algorithms	CTA	Vascular tree segmentation	Saxena et al. (2022) [22]	Single-centre, high classification precision
ANN/ML models	Clinical/demographic	Complication/risk prediction	Formeister et al. (2020) [17]; Asaad et al. (2023) [14]	Single-centre cohorts, up to ~95% predictive accuracy
LLM (ChatGPT)	Text FAQ queries	Patient education clarity/completeness	Jeong et al. (2023) [18]	Single-centre survey, ChatGPT: ~92% accuracy
Attention-UNet	Preop imaging	Microvascular perforator detection	Shen et al. (2022) [12]	Single-centre, outperforms manual selection
BreastGAN (generative AI)	Preop photos	Simulated aesthetic outcomes	Chartier et al. (2022) [16]	Single-centre comprehension study
HSI + CNN	Hyperspectral images	Flap malperfusion detection	Maktabi et al. (2025) [36]	Early pilot, promising sensitivity/specificity
CUSUM + ML	Intraop metrics	Quality/success benchmarking	Tighe et al. (2022) [41]	Single-centre performance deviations detection

## Abbreviations

The following abbreviations are used in this manuscript:

AI	Artificial Intelligence
ML	Machine Learning
LLM	Large Language Model
DIEP	Deep Inferior Epigastric Perforator
CTA	Computed Tomography Angiography
SSIM	Structural Similarity Index
PSNR	Peak Signal-to-Noise Ratio
GAN	Generative Adversarial Network
CNN	Convolutional Neural Network
ALT	Anterolateral Thigh
H&N	Head and Neck
AR	Augmented Reality
ICG	Indocyanine Green
ANN	Artificial Neural Network
HSI	Hyperspectral Imaging
ICU	Intensive Care Unit
CUSUM	Cumulative Sum (Control Chart)
MeSH	Medical Subject Headings
EMTREE	Elsevier Life Science Thesaurus
CENTRAL	Cochrane Central Register of Controlled Trials
